# Human *ROBO1* regulates white matter structure in corpus callosum

**DOI:** 10.1007/s00429-016-1240-y

**Published:** 2016-05-30

**Authors:** Fahimeh Darki, Satu Massinen, Elina Salmela, Hans Matsson, Myriam Peyrard-Janvid, Torkel Klingberg, Juha Kere

**Affiliations:** 1 Department of Neuroscience, Karolinska Institutet, Stockholm, Sweden; 2 Research Programs Unit, Haartman Institute, University of Helsinki, Helsinki, Finland; 3 Folkhälsan Institute of Genetics, Helsinki, Finland; 4 Department of Biosciences and Nutrition, Karolinska Institutet, Hälsovägen 7, 14183 Huddinge, Sweden; 5 Science for Life Laboratory, Karolinska Institutet, Solna, Sweden

**Keywords:** Structural MRI, DTI, Single nucleotide polymorphism, Roundabout, Axon guidance receptor homolog 1, Axonal pathfinding

## Abstract

The axon guidance receptor, Robo1, controls the pathfinding of callosal axons in mice. To determine whether the orthologous *ROBO1* gene is involved in callosal development also in humans, we studied polymorphisms in the *ROBO1* gene and variation in the white matter structure in the corpus callosum using both structural magnetic resonance imaging and diffusion tensor magnetic resonance imaging. We found that five polymorphisms in the regulatory region of *ROBO1* were associated with white matter density in the posterior part of the corpus callosum pathways. One of the polymorphisms, rs7631357, was also significantly associated with the probability of connections to the parietal cortical regions. Our results demonstrate that human *ROBO1* may be involved in the regulation of the structure and connectivity of posterior part of corpus callosum.

## Introduction

The corpus callosum (CC), a white matter structure found only in placental mammals, is the largest axon tract in the brain. Within the CC, the axons from callosal projection neurons cross the cortical midline, connect the two cerebral hemispheres, and are involved in many higher cognitive functions by transferring information between the hemispheres (Fame et al. 2011).

During the development of the CC, the extension, guidance, and contralateral targeting of the callosal axons are tightly regulated. Axonal crossing of the midline of the callosal projection neurons is guided by long-range (secreted) and short-range (membrane-associated or transmembrane) guidance cues provided most importantly by glial cell populations in the midline structures (Fame et al. 2011). One of the transmembrane receptors that is reported to be involved in callosal axon guidance is the roundabout homolog 1 (*Robo1*) receptor, which is present in callosal axons at high levels as they approach and cross the midline during mouse emryonal development (Shu et al. 2003). The ligands for the Robo1 receptor belong to the Slit family of chemorepulsive guidance cues, of which Slit2 appears to be most important in callosal development. (Andrews et al. 2006; López-Bendito et al. 2007; Unni et al. 2012). Robo1 also participates in the guidance of other major axonal projections in the forebrain (Andrews et al. 2006, Lopez-Bendito et al. 2007).

Homozygous *Robo1* knock-out mice (*Robo1* ^−/−^) that express no *Robo1* mRNA or protein die at birth and display severe dysgenesis of the CC (Andrews et al. 2006, Unni et al. 2012), whereas a homozygous *Robo1* mutant mouse strain with very little expression of a mutant allele of *Robo1* was viable (Long et al. 2004) and displayed only minor defects in the pathfinding of callosal axons (López-Bendito et al. 2007). In the *Robo1* ^−/−^ mice, the misrouted callosal axons terminate in the septum without crossing the midline (Andrews et al. 2006; Unni et al. 2012), whereas those that crossed the midline form normal homotopic connections in the contralateral hemisphere (Unni et al. 2012).

Besides the role of *Robo1* in axon guidance, the inhibition of Robo1-mediated signaling has been reported for its role in proliferation and migration of neocortical interneurons (Andrews et al. 2006, 2008; Hernández-Miranda et al. 2011). Robo1 has also been found to regulate the proliferation and generation of pyramidal neurons (Yeh et al. 2014) and their migration to layers II/III in the neocortex (Gonda et al. 2013). In mice, there are four homologous genes in the roundabout receptor family, of which *Robo1*, *Robo2,* and *Robo3* are expressed in the central nervous system and also in other tissues, such as the adrenal gland, limbs, and eye, respectively, whereas *Robo4* expression is limited mostly to endothelial cells (the FANTOM Consortium 2014).

The precise role of *ROBO1* in the development of the human CC is unknown, but *ROBO1* has been implicated in cognitive functions related to language, which require efficient communication between the hemispheres. The earliest observations were from a large Finnish family, in whom a rare haplotype in the genomic area of *ROBO1* was coinherited with developmental dyslexia and was associated with reduced levels of *ROBO1* expression in lymphoblasts. Moreover, in an unrelated dyslexic individual, a translocation breakpoint was found to disrupt *ROBO1* (Hannula-Jouppi et al. 2005). More recently, ROBO1 was associated with developmental dyslexia in Canadian family based analysis, with the dyslexia-associated allele also correlating with low gene-expression in brain tissue (Tran et al. 2014). *ROBO1* has also been suggested to have a specific function in supporting a short-term buffer for arbitrary phonological strings, which is an important trait during language acquisition (Bates et al. 2011).

Within the normal population, the expression of *ROBO1* is known to vary (Spielman et al. 2007), most probably reflecting genetic variation in the *ROBO1* locus. Numerous studies have reported inter-individual variability in the CC structure (Luders et al. 2010; Paul 2011; Hasan et al. 2012; Vandermosten et al. 2012, 2013). We hypothesized that if *ROBO1* controls midline crossing of callosal axons in humans, we might see the association of *ROBO1* single nucleotide polymorphisms (SNPs) with CC structure. Moreover, since *ROBO1* has been found to regulate the migration and laminar distribution of pyramidal neurons in mice, we tested the effect of *ROBO1* SNPs on the thickness of cortex.

## Materials and methods

### Participants

Structural magnetic resonance imaging and diffusion-weighted imaging were carried out on 76 typically developing children and young adults, aged between 6 and 25 years. The participants were randomly selected from a longitudinal study (three time-points, each 2 years apart), and they were all from the population registry in Nynäshamn, Sweden (Söderqvist et al. 2010). The study was approved by the local ethics committee of the Karolinska University Hospital, Stockholm, Sweden. Informed consent was obtained from all individual participants included in the study. The informed consent was provided by the parents of children aged below 18 years.

### Genotyping and quality control steps

Genotyping was performed on the Affymetrix Genome-wide Human SNP array 6.0, including more than 906,600 SNPs and more than 946,000 probes for detecting copy number variation. The genotyping was done on one batch with DNA extracted from blood (*n* = 63) and two batches with DNA extracted from saliva (*n* = 19 and *n* = 8) using standard methods and commercial kits. Two individuals were removed from the blood batch in initial quality control. DNA from these individuals was reextracted from saliva, and genotyping was redone using saliva DNA.

The genotypes were called using Birdsuite version 1.5.5 (Korn et al. 2008) separately on each batch. Conversion of the output files to PLINK-compatible format was done using the Birdsuite to PLINK pipeline version 1.6.6. (https://www.broadinstitute.org/ftp/pub/mpg/birdsuite/Birdsuite_Pipeline.pdf). The reference genome assembly used was hg18.

Quality control was done using PLINK version 1.07. (Purcell et al. 2007) and conducted on two levels: exclusion of individuals and exclusion of SNPs. No individuals were removed due to low genotype call rates. The average genotype call rate of individuals was over 98 % in all of the batches. In total, 13 individuals were removed from the analysis based on either gender discordance detected using heterozygosity rates of X-chromosomal SNPs (one individual), excessive heterozygosity detected by calculating inbreeding coefficients (three individuals), possible cryptic relatedness estimated by pairwise identity-by-state analysis (two individuals), or known relatedness (seven individuals). Out of the known and suspected sib-pairs, the sibling with a larger proportion of missing genotypes was removed from the analysis.

Before quality control, 909,622 SNPs were available for analysis. SNPs with a proportion of missing genotypes >0.05 or with a missing genotype for more than one individual in any batch were removed from all batches (48,451 SNPs in total). Also, the following SNPs were removed: 137753 SNPs with minor allele frequency (MAF) <0.01, 26 SNPs showing deviation from Hardy–Weinberg equilibrium (*p* < 1 × 10^−5^), 39 SNPs showing non-random missingness with respect to neighboring genotype and 37 SNPs showing association with batch membership. Overall, 79.5 % (723,316/909,622) of the markers passed all quality control filters.

After filtering, 76 individuals (41 males and 35 females) and 723,316 diploid SNPs remained. The average individual call rate was 99.7 %, and the lowest individual call rate was 97.4 %.

### Selection of *ROBO1* SNPs

From the quality-controlled genotype data, we first selected all SNPs from the genomic interval chr3:78,720,000–80,000,000 (hg18), in total 201 SNPs. This area encompasses all of the annotated transcript variants of the *ROBO1* gene (NM_002941.3, NM_133631.3 and NM_001145845.1) as well as roughly 300- and 10-kb upstream of the longest variant (NM_002941.3).

A subset of these SNPs was chosen for a two-phase association study; in the first phase, we selected tagging SNPs to efficiently capture the common genetic variation in the area while keeping the multiple testing burden to a minimum, and in the second phase, we refined the association analysis by selecting additional, more closely spaced SNPs within the genomic locations that contained associated SNPs in the first phase.

In the first phase, Haploview (Barrett et al. 2005) was used to construct a haplotype map using the 201 SNPs in the genomic region of *ROBO1*. Pairwise comparisons of markers >1000 kb apart were ignored. The resulting 19 haplotype blocks were subjected to Haploview’s Tagger algorithm to find best tagging SNPs for them, with settings of the minor allele frequency (MAF) > 0.1 and a maximum number of tags to pick of =20. The selected 20 tagging SNPs (rs3773216, rs9875094, rs3773232, rs1457659, rs416551, rs7629522, rs162870, rs162871, rs162262, rs162429, rs7631406, rs12497294, rs6770483, rs9835692, rs9876238, rs4856291, rs4856447, rs12488868, rs6768880, and rs9830013) captured 98 alleles (58 % of the 169 alleles with MAF > 0.1) at *r* ^2^ ≥ 0.8 and 57 % of alleles with mean *r* ^2^ of 0.937.

In the second phase, we selected additional SNPs within and between the two haplotype blocks whose tagging SNPs (rs17396958 and rs1393375) had shown association in the first phase. We chose MAF >10 % as cutoff. The SNPs that did not show association in the first phase and the SNPs tagged by them were excluded. Of those SNPs that were in perfect linkage disequilibrium (LD) with each other, only one SNP was selected. Between rs17396958 and rs1393375, we also dropped out all but one SNP per group from groups of SNPs that were in strong LD with each other (*r* ^2^ > 0.8). In total, there were 28 SNPs analyzed in the second phase: rs6770755, rs7651370, rs7631357, rs4564923, rs6548621, rs9832405, rs7637338, rs6548628, rs9853895, rs9820160, rs7432676, rs9309825, rs13071586, rs13072324, rs6771681, rs7618126, rs7432306, rs6548650, rs7644521, rs1995402, rs17380584, rs11917376, rs11706346, rs1393360, rs1502298, rs10511118, rs1502305, and rs10511119.

### Structural brain imaging and voxel-based morphometry

We applied three-dimensional magnetization prepared rapid gradient echo (MP-RAGE) sequence with TR = 2300 ms and TE = 2.92 ms to collect structural MRI data with 256 × 256 mm^2^ field of view (FOV), 256 × 256 matrix size, 176 sagittal slices, and 1-mm^3^ isotropic voxel size. The structural images were then processed using Diffeomorphic Anatomical Registration Through Exponentiated Lie Algebra (DARTEL) method, which segmented the brain into gray matter, white matter, and cerebral spinal fluid.

DARTEL, as a part of SPM5 software, was performed on structural data collected at all three time-points, and white matter was segmented after all images were registered to the template generated by iterative registration of all individuals’ T1-weighted images. To preserve the total amount of signal from different regions in the brain, the modulation step was also performed. The modulated white matter segmented (white matter density) images were then smoothed with an 8-mm Gaussian kernel and fed into a higher level statistical analysis in SPM5 for detecting any SNPs associations with white matter structure.

### Diffusion tensor imaging

Diffusion tensor imaging (DTI), with the scanning parameters of 230 × 230 mm^2^ FOV, 128 × 128 matrix size, 40 slices with thickness of 2.5 mm, and b-value of 1000 s/mm^2^ in 64 gradient directions, was collected at the third round of longitudinal data collection (Söderqvist et al. 2010). Eddy current and head motions were corrected with affine registration for all diffusion-weighted images to a reference volume using FSL software (http://fsl.fmrib.ox.ac.uk/fsl/fslwiki/). The diffusion tensor parameters were then estimated for each voxel, and subsequently, the DTI and fractional anisotropy (FA) data were constructed. Non-linear registration was carried out using Tract-Based Spatial Statistics, TBSS v1.2, (http://fsl.fmrib.ox.ac.uk/fsl/fslwiki/TBSS) to align all FA images to the mean FA skeleton.

### Probabilistic fiber tracking of CC

To find the CC white matter fibers, the body of CC was selected as seed region and probabilistic tractography was performed on all individuals’ DTI data, initiating from all voxels within the seed masks using probtrackx tool of FDT v2.0, FSL (http://fsl.fmrib.ox.ac.uk/fsl/fsl-4.1.9/fdt/fdt_probtrackx.html). The fiber tracking parameters were set as default, 5000 streamline samples, step length of 0.5 mm, and curvature threshold of 0.2. At the individual level, the probabilistic connectivity maps were first normalized by dividing with the corresponding way total value, as the total number of generated tracts. The number of the generated tracts for each individual was related to the size of the seed (i.e., the body of CC). The normalized probability maps were then thresholded by 5 % of the samples to remove the voxels with low probability of connection (Leh et al. 2006). In the next step, all of the traced white matter pathways were aligned using the TBSS method for non-FA images and then binarized and averaged across all subjects. To define a mask of CC fibers, the group probability map of the tracts was finally thresholded at the group level by keeping the pathways that were present in 90 % of the cases. This mask was later used as a region of interest for small volume correction in higher level statistical analysis of the white matter structure in association with *ROBO1* SNPs.

### Probabilistic fiber tracking for segmenting CC

Five different cortical regions of interest, including anterior frontal, superior frontal, parietal, temporal, and occipital cortex, were selected bilaterally as target regions for probabilistic fiber tracking of CC to segment this large white matter tract to smaller segments. These cortical regions were defined based on the Harvard–Oxford cortical atlas, and the body of CC was considered as the seed region for initiating the fiber tracking.

In the next step, the white matter fibers found from all five regions of interest were thresholded by 5 % of the maximum values to exclude the tracts with the probability of connections lower than the threshold. After segmenting the CC pathways based on the probabilistic fiber tracking of the CC, the averages of two different indices were computed in these five segmented white matter tracts. The first index was the probability of connection to each cortical region, which indicates the structure of white matter pathways (such as number, thickness, size, and the myelination of axons). The second one was FA that reflects the organization and packing of the axons as well as myelination.

### Cortical thickness measurements

To assess the association of *ROBO1* SNPs with thickness of cortex, the cortical thickness of the structural MRI data was computed using automatic longitudinal stream in FreeSurfer (Reuter et al. 2012). All structural data were first registered to a within-subject template (Reuter et al. 2010; Reuter and Fischl 2011). After applying several processing steps (Dale et al. 1999; Fischl and Dale 2000), including skull removing, template transformation, and atlas registration, the images were later segmented to white matter, gray matter, and pial based on intensity and neighborhood voxel restrictions. Thickness of cortex was computed as the distance between the white matter and the pial. The cortical thickness of the cortical regions of interest (including the left and right anterior frontal, superior frontal, parietal, temporal, and occipital) was then calculated using the workflow described in http://surfer.nmr.mgh.harvard.edu/fswiki/VolumeRoiCortical Thickness.

### Statistical analyses

The white matter segmented images were analyzed by higher level SPM analysis, using a flexible factorial design (http://www.fil.ion.ucl.ac.uk/spm/software/spm8), to assess the association of *ROBO1* SNPs with white matter structure. In the first phase of assessment, all 20 SNPs were entered separately as a main factor in the model. Subjects and testing time-points were also considered as factors in the flexible factorial model to consider the repeated measures. Age, gender, handedness, and total white matter volume were used as covariates, and the interactions of SNP, as the main factor, with age and gender were also added. Two of the SNPs (rs17396958 and rs1393375) were significantly associated with white matter density in the posterior part of the corpus callosum. In the second phase, we tagged 28 SNPs within and between the two haplotype blocks of these two SNPs. Then, the exploratory analysis was done within the white matter masked by the group probability map of the CC tracts, with non-stationary cluster extent correction, at FDR-corrected cluster level (*p* value of 0.05). We corrected for multiple comparisons (Bonferroni correction) of the number of SNPs (28 SNPs) and, accordingly, set the threshold of significant *p* values at 0.0018.

After fiber tracking and segmenting the CC into five parts, the mean value of two indices (probability of connection and FA) was computed in these five segments. These measures were then analyzed for associations between these brain measures and the SNPs significantly associated with white matter density within CC in the second phase of the analysis using linear regression in IBM SPSS statistics 21.0 software. For each test, brain measures were included as dependent variable, and age, gender, handedness, and genotypes were included as independent variables.

The same linear regression analysis were also performed for the measures of cortical thickness in all five cortical regions of interest using the thickness measures as the dependent variable and similar independent variables as mentioned above.

## Results

### Five *ROBO1* SNPs associated with white matter structure in the CC

We studied the hypothesis whether common SNPs anywhere within the *ROBO1* gene are associated with morphological variation of the CC. Because selecting tagging SNPs for association studies can be a tradeoff between efficiency and power (more SNPs would capture more variation but might result in less power to detect subtle genetic effects because of the burden of multiple testing), we chose to perform the analysis in two phases. The first phase asked whether anywhere within the ROBO1 genomic region, there would be common variants associating with CC variation. In the second phase, we seeked confirmation and refinement by additional SNPs for those genomic regions that had passed the initial test of association.

In the first phase, 20 tagging SNPs in the genomic region of the *ROBO1* gene were chosen for voxel based analysis on white matter segmented images. Two of the SNPs (rs17396958 and rs1393375) were significantly associated with white matter density in posterior part of CC (*p* = 5.4 × 10^−6^ and *p* = 2.02 × 10^−5^, respectively, at FDR-corrected cluster level of *p* value <0.05). The associated SNPs are both located upstream of the *ROBO1* transcription start site, possibly in regulatory regions.

**Fig. 1:**
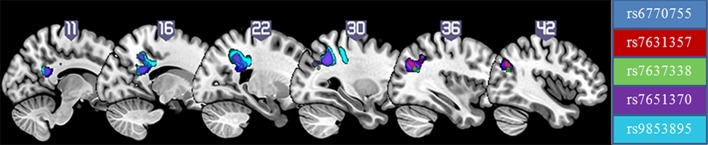
Five SNPs showed significant effect on white matter density in the right posterior part of the CC

Because the 20 SNPs covered less than 50 % of single nucleotide variation in the *ROBO1* locus, we then attempted to confirm and refine the genetic association by selecting more SNPs between and within the haplotype blocks that associated with CC structure in the first phase, and then repeated the analysis with the new SNPs. In the second phase of the association analysis, five of the 28 SNPs (Fig. 1) showed a significant effect on white matter density in the right posterior part of CC, connecting the parietal and occipital cortical regions. All significant clusters overlapped with each other (*p* value at the FDR level, correcting for multiple comparison of 28 SNPs, *p* < 0.0018). Table 1 lists the peak coordinates, the cluster size and the *p* value of the clusters significantly associated with each of those five SNPs.

**Table 1:** Coordinates of the peak voxels for each cluster associated with each SNP

SNP	P_FDR-corrected_ cluster-level	Cluster size	Peak voxel	
			*Z*	*x*, *y*, *z* (MNI)
rs6770755	5.49 × 10^−5^	1701	6.43	37, −66, 30
rs7631357	1.40 × 10^−3^	130	5.13	41, −65, 30
rs7637338	3.89 × 10^−4^	78	4.58	42, −65, 25
rs7651370	6.40 × 10^−5^	1326	6.03	37, −66, 30
rs9853895	4.89 × 10^−5^	2182	5.79	29, −52, 38

**Fig. 2:**
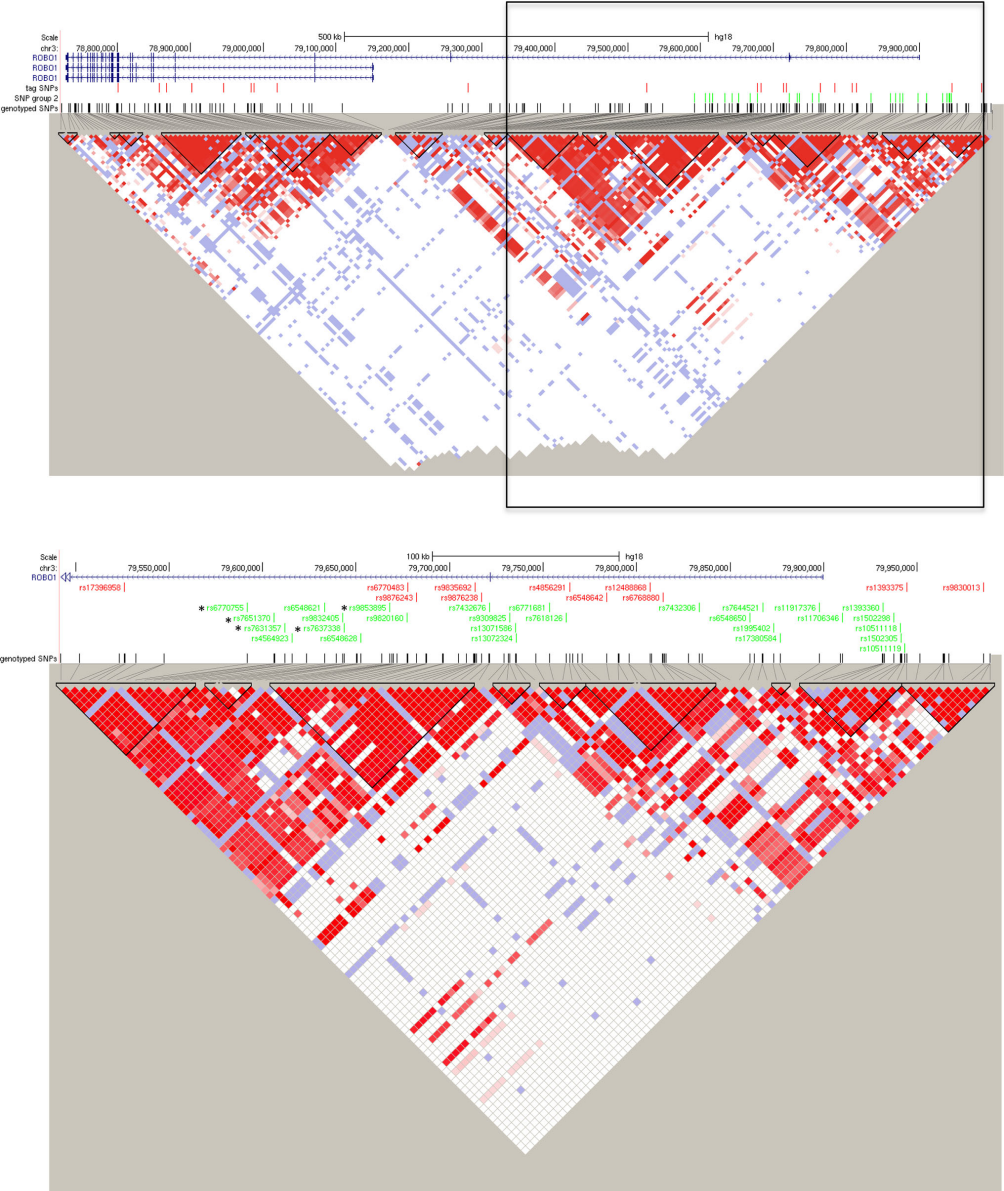
Genomic extend of *ROBO1* on the minus strand of chromosome 3 (*top*) and magnified view of the focus of the second round of genotyping (*bottom*). The magnified area is marked with a black rectangle on the uppermost LD plot. The SNPs found in the quality controlled genotype data set are *marked in black*. The tagging SNPs selected in round one are marked in red, and the round 2 SNPs are marked in green. The SNPs that were associated with white matter density in the CC are *marked with asterisks*. The LD plot was constructed using Haploview and the19 haplotype blocks are displayed as *black triangles*. The three ROBO1 transcript variants (NM_002941.3, NM_133631.3 and NM_001145845.1) were extracted from UCSC genomic browser. Genomic coordinates are marked according to GRCh37/hg19

The five associated SNPs are located in the second intron of the longest transcript variant (NM_002941.3) of *ROBO1*, and are therefore potential sites for the regulation of *ROBO1* expression. The SNPs appear to belong to an extended haplotype block, although Haploview software has further divided it into three smaller blocks (Fig. 2).

The BrainCloud database contains information on the temporal dynamics and genetic control of transcription in human prefrontal cortex (Colantuoni et al. 2011). In this database, only one SNP (rs331142) in the *ROBO1* gene showed a trend for association to ROBO1 expression (*p* = 4.6 × 10^−5^), but it did not reach genome-wide statistical significance (Tran et al. 2014). Rs33114 is not found on the Affymetrix Genome-wide Human SNP array 6.0 that was used in the genotyping in our study and is located roughly 600 kb from the haplotype block containing the five SNPs associated with white matter density in our study.

### rs7631357 correlates with the probability of connection to parietal areas

**Fig. 3:**
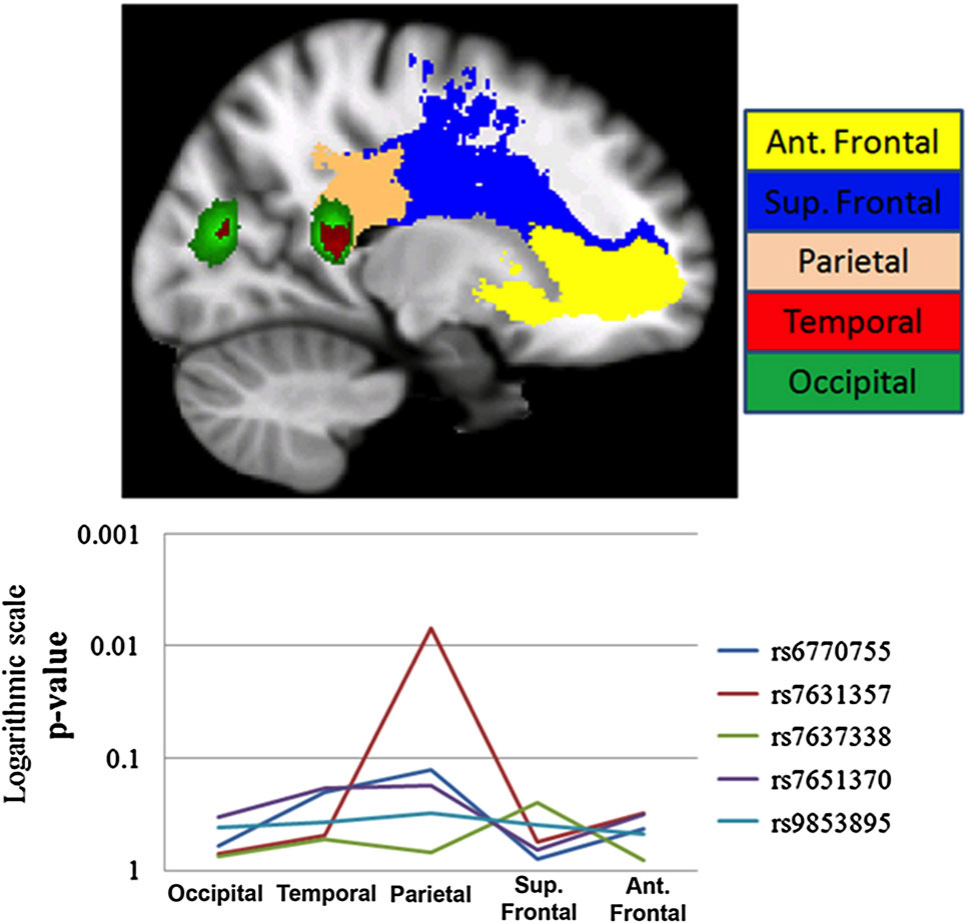
**a** Five different segments of CC segmented by the probabilistic fiber tracking of the body of CC with connections to the anterior frontal, superior frontal, parietal, temporal and occipital cortex, bilaterally. **b** Logarithmic scale of the *p* values for the associations between five significant SNPs and the probability of connection in five different segments of the CC (shown by* different colors* in a sagittal section of the brain)

Two different indices (probability of connection and FA) computed in the five segmented white matter tracts (shown in Fig. 3a) were analyzed for associations between these measures and the five SNPs significantly associated with white matter density within CC. After correction for multiple comparisons (*p* < 0.002, Bonferroni correction for 25 tests), the mean probability of connection of the body of the CC to the parietal areas significantly correlated with one of the SNPs, rs7631357 (*p* = 1.09 × 10^−5^). The mean FA values in the traced pathways did not correlate with the genotype variations in any of these regions. The logarithmic graph shows the significant level of the association of each SNP and the probabilities of connections to different regions of interest (Fig. 3b).

In the assessment of the association of *ROBO1* SNPs to cortical regions of interest in both the left and right hemispheres, three SNPs (rs6770755, *p* = 0.015; rs7631357, *p* = 0.026; rs7651370, *p* = 0.009) showed a trend for association with cortical thickness of the left parietal region, although none of the associations remained significant after correction for multiple comparisons for 50 tests. We also assessed the interhemispheric asymmetry by the asymmetry coefficient (AC = 100 × (*L*_CT_ − *R*_CT_)/[(*L*_CT_ − *R*_CT_)/2]) of the thickness of the five cortical regions of interests. L_CT_ and R_CT_ are the cortical thickness in the left and right hemisphere, respectively. We tested the association of the five *ROBO1* SNPs with the AC. Four SNPs showed significant associations to AC of parietal region (rs6770755, *p* = 3.3 × 10^−4^; rs7631357, *p* = 9.2 × 10^−5^; rs7651370, *p* = 0.003; rs9853895, *p* = 3.29 × 10^−4^), and all *p* values survived the multiple comparisons. The AC of other cortical regions did not show any significant correlations with the SNP variations (all *p* values >0.05). In all of the above analyses, we corrected for the effect of age, sex, and handedness, though none of the covariates were significant. The significant associations of the *ROBO1* SNPs were in agreement with white matter associations in posterior part of CC with connections to parietal cortex.

## Discussion

The association of *ROBO1* SNPs with white matter structure was assessed using two white matter structural measures (white matter density and probability of connections). We found that five SNPs in the presumed regulatory region of *ROBO1* were associated with white matter density in the posterior part of the CC.

Robo1 has been implicated in axonal pathfinding in mice: in *Robo1* ^−/−^ knock-out mice, callosal axons were found to be misrouted. In our study, the participants were normal, typically developing children and young adults. We had no RNA samples from them for measuring *ROBO1* expression. In this study, we were able to correlate the variations in *ROBO1* SNPs with white matter structure in the CC. SNP rs7631357 significantly correlated with both the white matter density in the posterior part of CC and the probability of connections from the body of CC to parietal regions. These findings fit well with previous observations of the role of Robo1 in axonal pathfinding in mice (Andrews et al. 2006), although it should be mentioned that the white matter indices do not directly explain the biophysical factors such as size, diameter, membranes, myelin thickness, or packing of axons.

The posterior part of CC interconnects the temporal, parietal, and occipital cortices with large and heavily myelinated axons (Aboitiz et al. 1992). Although morphological and shape analysis studies have reported some inconsistent findings, the posterior CC has been reported larger in dyslexic subjects compared with normal readers in some CC shape analysis studies (Duara et al. 1991; Rumsey et al. 1997; Duta 2000; Hasan et al. 2012). The microstructure of posterior CC, such as FA, has also been associated with reading skills and has been found higher in dyslexic compared with typically developing readers (Frye et al. 2008).

Previously, SNPs within three dyslexia candidate susceptibility genes (*DYX1C1*, *DCDC2*, and *KIAA0319*) have been shown to be associated with white matter density in the left temporoparietal region. Moreover, the white density was positively correlated with reading ability (Darki et al. 2012). The white matter regions associated with the *ROBO1* SNPs partially overlapped with the white matter area found in association with rs6935076 in *KIAA0319* (Darki et al. 2012).

*ROBO1* has previously been shown to affect axonal midline crossing of auditory pathways (Lamminmäki et al. 2012) in dyslexic cases from the rare Finnish family with reduced expression of *ROBO1* (Hannula-Jouppi et al. 2015). The midline crossing was studied using interaural interaction as a biomarker, as interaural interaction is dependent on axonal midline crossing present in the auditory pathways (Lamminmäki et al. 2012). Although axons within the CC connect the auditory cortices of the two hemispheres, the deficit in interaural interaction is thought to be dependent on axonal crossing occurring possibly at multiple stages at the afferent central auditory pathway passing through the brainstem, the midbrain, and the thalamus, ending up at the auditory cortex. Thus, our findings about the possible role of ROBO1 in callosal development are separate from the earlier findings on the role of ROBO1 in axonal crossing of the auditory pathways, and our results are first to suggest that *ROBO1* may also regulate the midline crossing of callosal axons in humans. In contrast to the previous studies of *ROBO1* in the single Finnish family (Lamminmäki et al. 2012), our results link for the first time the *ROBO1* gene to a specific structural feature of the human brain in the general population.

We also studied whether the *ROBO1* SNPs would be associated with cortical thickness as well as the interhemispheric asymmetry of the cortical thickness. Three SNPs showed a trend toward association to the cortical thickness in the left parietal cortex. Four of the SNPs showed significant associations with the asymmetry coefficient of the parietal cortex, which fits well with previous observations of *Robo1* controlling neocortical lamination in mice (Gonda et al. 2013). Taken together, our results suggest that similar to *Robo1* in mice, the human *ROBO1* is likely to have various roles in axonal pathfinding and neuronal migration during brain development, which may lead to asymmetry of both white matter and gray matter in connection with CC.

Interestingly, the *ROBO1* SNPs associating with the structural measures in CC and cortex are localized to the most proximal third of the gene, i.e., the region that harbors several correlated minor transcription start sites for *ROBO1*. These promoters (annotated by the FANTOM5 study as p4, p6, p9, p11, and p27) are associated with *ROBO1* expression especially in the fetal temporal, parietal and occipital lobes (the FANTOM Consortium 2014). This expression pattern agrees overall with a role of the genomic region harboring the associated SNPs in regulating axon growth during the fetal period and thus structural correlates even after birth.

The sample size used in our study is relatively small, and consequently, a replication in larger data sets may further refine the observed genetic regulatory effect. Overall, the results demonstrate that variability in the human *ROBO1* locus may contribute to the variability in the structure and connectivity of posterior part of CC, and is likely to have functional relevance in the transfer of information between the hemispheres.
